# Once a year school-based deworming with praziquantel and albendazole combination may not be adequate for control of urogenital schistosomiasis and hookworm infection in Matuga District, Kwale County, Kenya

**DOI:** 10.1186/1756-3305-7-74

**Published:** 2014-02-19

**Authors:** Sammy M Njenga, Faith M Mutungi, Claire Njeri Wamae, Mariam T Mwanje, Kevin K Njiru, Moses J Bockarie

**Affiliations:** 1 Eastern and Southern Africa Centre of International Parasite Control (ESACIPAC), Kenya Medical Research Institute (KEMRI), Mbagathi Road, Nairobi, Kenya; 2 Centre for Microbiology Research (CMR), Kenya Medical Research Institute (KEMRI), Mbagathi Road, Nairobi, Kenya; 3 Division of Communicable Disease Prevention and Control, Ministry of Health, Nairobi, Kenya; 4 Kwale District Hospital, Kwale County, Kenya; 5 Centre for Neglected Tropical Diseases, Liverpool School of Tropical Medicine, Liverpool, UK

**Keywords:** School-based deworming, Praziquantel, Albendazole, Urogenital schistosomiasis, Soil-transmitted helminths, Kenya

## Abstract

**Background:**

Neglected tropical diseases (NTDs) predominantly occur in resource poor settings where they often present a serious public health burden. Sustained global advocacy has been important in raising awareness of NTDs and the relatively low cost for control of helminthic NTDs using preventive chemotherapy. This enthusiasm was boosted at the London declaration on NTDs in 2012 through commitments by different partners to avail resources required for control of NTDs particularly those that employ preventive chemotherapy as the major intervention strategy. Subsequently, national NTD programmes are responding to these new opportunities by implementing preventive chemotherapy including school-based deworming (SBD). Further, with the availability of increased resources, both financial and pharma, the optimal strategies for implementing preventive chemotherapy in highly endemic settings are under debate and this paper goes some way to addressing this issue in a specific setting in coastal Kenya.

**Methods:**

We conducted a repeated cross-sectional study in Matuga District, Kwale County, Kenya to evaluate the effect of school-based co-administration of praziquantel and albendazole against urogenital schistosomiasis and soil-transmitted helminth (STH) infections. A total of 1022 school children in 5 study schools were tested for the infections in urine and stool samples during a baseline survey in September 2009. The presence of *Schistosoma haematobium* infection was determined by the urine filtration method while STH infections were determined by Kato-Katz technique.

**Results:**

Urogenital schistosomiasis and hookworm infection were the major parasitic infections among the children in the study area. There was significant decrease in both prevalence and intensity of *S. haematobium* infection after treatment but varying levels of rebound were observed during the period between the treatments. The school-based treatment, however, did not have any significant effect on both the prevalence and intensity of hookworm infection.

**Conclusions:**

Once per year SBD programmes may not be adequate for controlling hookworm infection and urogenital schistosomiasis in rural areas of Kwale County. There is a need to consider expanded preventive chemotherapy strategies that will allow inclusion of the adult populations. Community-based health education campaigns focusing on increasing household latrine ownership and use, as a complementary measure to control STH and urogenital schistosomiasis in similar settings, may also be useful.

## Background

Neglected Tropical Diseases (NTDs) are a group of parasitic and bacterial infections that are strongly associated with disability, disfigurement, stigma and poverty. The World Health Organization (WHO) currently focuses on 17 NTDs including parasitic helminths [1] which cause chronic infections. In many developing countries parasitic helminths remain a significant public health problem, particularly in communities with low levels of education, inadequate sanitation and lack of clean and safe water.

Soil-transmitted helminth (STH) infections are caused by gastrointestinal nematodes (also known as geohelminths), namely *Ascaris lumbricoides*, hookworms (*Necator americanus* and *Ancylostoma duodenale*), and *Trichuris trichiura* and are among the most prevalent human infections in the world. It is estimated that around 1.4 billion individuals are infected by *A. lumbricoides*, 1.0 billion by *T. trichiura*, and 1.3 billion by hookworms [2]. Schistosomiasis (bilharzia) is a highly focal disease caused by blood trematodes of the genus *Schistosoma*. Most human infections are caused by *Schistosoma mansoni, S. haematobium* or *S. japonicum*, with two other species (*S. intercalatum* and *S. mekongi*) contributing less to the case load [3]. An estimated 200 million people in 74 countries have schistosomiasis, 85% of whom live in sub-Saharan Africa where *S. haematobium*, *S. mansoni* and *S. intercalatum* are endemic [4]. Humans are infected during contact with water infested with larval forms of the parasite called cercariae released by intermediate freshwater snail hosts. Both STH and schistosomiasis infections are highly prevalent in most areas of East Africa [5].

Chemotherapy has an important role not only in the treatment of individual patients but also, in conjunction with public health and vector control measures, in reducing the transmission of parasitic infections [6]. A call was made in 2001 at the Fifty-fourth World Health Assembly, when WHO Member States were urged to ensure provision for regular administration of anthelminthic chemotherapy to at least 75% of school-age children as a target for the control of morbidity due to STH and schistosomiasis [2]. Consequently, many endemic countries have implemented school-based deworming (SBD) programmes as a preventive chemotherapy strategy to control STH and schistosomiasis. Kenya launched a SBD programme in 2009 and received additional support in 2011 from partners to sustain and scale up the programme [7].

On 30 January 2012 during what is referred to as the London declaration on NTDs, several pharmaceutical companies made new commitments to sustain, expand and extend donations to ensure the necessary supply of drugs and other interventions to help in the control of NTDs. The donations include the anthelminthic drugs albendazole and mebendazole by GlaxoSmithKline and Johnson & Johnson respectively plus the anti-schistosomal drug praziquantel by Merck KGaA. To be able to obtain the best outcomes possible with these increased resources to combat NTDs, it is critical to determine the most optimal strategies for preventive chemotherapy in different epidemiological settings. The aim of the current study was to determine the effectiveness of once per year SBD against schistosomiasis and STH infections in school-age children living in 5 rural villages in an area where endemicity of both infections is high and under irregular mass drug administration (MDA) consisting of albendazole plus diethylcarbamizine (DEC) against lymphatic filariasis (LF).

## Methods and study area

### Study design

This study was designed to evaluate the effect of co-administration of praziquantel and albendazole against urogenital schistosomiasis and STH infections in an endemic area undergoing MDA for LF since 2003, albeit not given every year as recommended by the WHO. The primary study design in each community was school-based repeated cross-sectional surveys to estimate changes in parasitological variables (prevalence and intensity). The study protocol was reviewed and approved by the Scientific Steering and Ethical Review Committees of the Kenya Medical Research Institute (KEMRI).

### Study area and population

**Figure 1:**
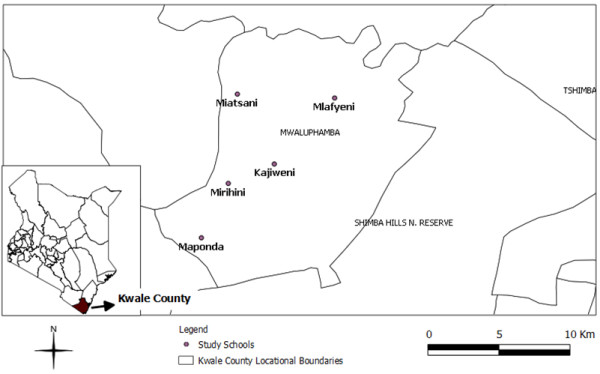
**Map of study area.** A map showing the location of the five study schools in Mwaluphamba Location, Matuga District, Kwale County, Kenya.

The study was conducted in Mwaluphamba Location, Matuga District in Kwale County in South Coast, Kenya an area where both STH and urogenital schistosomiasis are highly endemic. The details of the study area were previously described in a related report [8]. Figure 1 is a map showing the location of the study schools. Under the devolved government structure Kwale County consists of 4 districts, namely Msambweni, Lungalunga, Matuga and Kinango.

### Sampling methodology

A total of 5 study schools were randomly selected from public schools in Kizibe and Mlafyeni Sub-locations of Mwaluphamba Location in Kwale County. Enrolment of study participants targeted 200 children in each school attending classes 2 to 5 (aged 7-12 years). For each class, 50 children were randomly selected using a simple arithmetic systematic sampling procedure from the class registers provided by the class teachers. However, additional children from classes 6 to 8 were enrolled when 200 children could not be enrolled from target classes 2-5. Written consents were obtained from both the teachers and parents/guardians of the children. However, only two of the five study schools were selected for the final post-treatment parasitological survey in June 2012.

### Laboratory examinations

On the day prior to sample collection, registered study children were given stool containers, papers and wooden spatulas and instructed on how to collect a specimen of the morning stool. The following morning, the children were given containers for urine collection upon arrival of the research team at the school at around 9:00 to 10:00 am.

In a classroom provided by the school administration for specimen collection and processing, both stool and urine specimens were received and details of the children recorded. Stool specimens were labeled and stored in cool boxes with ice packs to lower the temperature hence avoiding any change of stool sample and hatching of the STH ova. The urine specimens were visually examined for blood and thereafter tested for microhaematuria, leukocyturia, and proteinuria using urinalysis reagent strips (ACON Laboratories, Inc., San Diego, CA, USA). Finally, a 10-ml volume of the urine was filtered through 25 mm diameter 12 μm polycarbonate filters (Nuclepore) and the filters placed on microscope glass slides for examination and counting of *S. haematobium* eggs.

The stool samples were transported to KEMRI field station laboratory located at the Kwale district hospital for processing and examination for STH ova. Briefly, a small amount of faecal material was sieved through a piece of screen to remove debris. Thereafter, duplicate Kato-Katz thick smears were prepared from each stool sample, using standard 41.7 mg templates and covered with cellophane strips pre-soaked in glycerol-malachite green solution. The slides were examined for ova and for positive samples the eggs counted and multiplied by 24 to be able to express the counts as eggs per gram of stool. All the slides were first examined for hookworm ova within 30-45 minutes of preparation and thereafter for other STH ova in a second round of examination. To ensure consistency of data the same team of technicians examined urine and stool specimens from all 5 study schools. For quality control, 10% of the urine filtration and Kato-Katz slides were re-read by the most experienced laboratory technologist.

### Timing of school-based deworming and parasitological surveys

Table 1 provides a timeline indicating the month and year when the treatments and parasitological surveys were conducted during the study. Briefly, a baseline survey was conducted in September 2009. The first round of SBD consisting of co-administration of praziquantel and albendazole was conducted in July 2010 by trained school teachers. Before the SBD, a one-day training of two teachers from each school in the location was conducted in a centrally located school. Distribution of praziquantel dose poles, tablets and treatment record forms was done after the training sessions. Supervisory visits were carried out in all the 5 study schools as well as a few other non-study schools on the treatment day. The first follow-up survey was undertaken in October 2010 (3 months after the first round of SBD). Additionally, a survey of adverse events experienced by the children after the first round of SBD was conducted in all the schools, the results of which have been published elsewhere [9]. A second follow-up survey was undertaken in March 2011 (8 months after first SBD). Thereafter, the National Programme for Elimination of LF (NPELF) conducted a community-wide mass drug administration (MDA) using DEC and albendazole in May 2011 (preceding round of MDA having been conducted in December 2008). The second round of SBD, using praziquantel and albendazole, was administered in January 2012 (6 months after the MDA against LF). The third follow-up survey was conducted in June 2012. Only two of the five study schools, however, were included in the third follow-up survey due to financial constraints. Mlafyeni school was selected because of the higher rate of *S. haematobium* infection at baseline whereas Mirihini was selected to represent the rest of the schools which had relatively higher hookworm infection rates compared with Mlafyeni. A third round of SBD again using praziquantel and albendazole was conducted in February 2013 by the national school deworming programme.

**Table 1:** Timeline summarizing the timing of parasitological surveys and deworming of children in study schools

**School**	**Sep**	**Jul**	**Oct**	**Mar**	**May**	**Jan**	**June**
	**2009**	**2010**	**2010**	**2011**	**2011**	**2012**	**2012**
**Kajiweni**	BLS	SBD1	FS1	FS2	CDT	SBD2	ND
**Maponda**	BLS	SBD1	FS1	FS2	CDT	SBD2	ND
**Miatsani**	BLS	SBD1	FS1	FS2	CDT	SBD2	ND
**Mirihini**	BLS	SBD1	FS1	FS2	CDT	SBD2	FS3
**Mlafyeni**	BLS	SBD1	FS1	FS2	CDT	SBD2	FS3

BLS: baseline survey; SBD1: school-based deworming 1; SBD2: school-based deworming 2; FS1: follow-up survey 1; FS2: follow-up survey 2; FS3: follow-up survey 3; CDT: Community-directed treatment with DEC and albendazole; ND: Not Done.

### Data analysis

The prevalence of the infections was expressed as the percentage of children found positive for *S. haematobium* and the three species of STH, namely *A. lumbricoides*, hookworms and *T. trichiura*. The intensities of the STH and schistosome infections were defined as number of eggs per gram (epg) of faeces and number of eggs counted in 10 ml of urine (eggs/10 ml), respectively. The Chi-squared test was used to test for linear association in the prevalences of schistosome and STH infections over the study period. The trends of arithmetic and geometric mean intensities were tested using simple regression analysis. Statistical significance was set at P < 0.05. The data analyses were performed using SAS (Statistical Analysis System, version 9.1, Cary, NC).

## Results

Table 2 summarizes the demographic characteristics of the children included in the study by year. Slightly more girls compared to boys participated in the surveys every year. A community demographic survey conducted at baseline in 1143 households in the five corresponding villages in the study area found that 896 (78.4%) of the households had no latrines and inhabitants practiced open defecation in the peridomestic areas.

**Table 2:** Demographic characteristics of children included in baseline and follow-up surveys for urogenital schistosomiasis and STH in Mwaluphamba Location, Mutuga District, Kwale County

	**Year and month of study**			
**Characteristics**	**Baseline**	**Oct**	**Mar**	**Jun**
	**(Sep 2009)**	**2010**	**2011**	**2012**
No. of children (n)	1022	1076	956	407
**Sex**				
Female, n (%)	555 (54.3%)	620 (57.6%)	564 (59.0%)	212 (52.1%)
Male, n (%)	467 (45.7%)	456 (42.4%)	392 (41.0%)	195 (47.9%)
**Age (years)**				
Median	12	12	12	11
Mean	11.6	11.7	12.4	10.8
Range	11	11	14	12
**No. of children tested in each study school**				
Kajiweni	197	206	211	ND
Maponda	200	221	187	ND
Miatsani	226	212	195	ND
Mirihini	200	223	214	196
Mlafyeni	199	214	149	211

ND: not done.

Treatment coverage was computed from specific treatment forms used to record the number of children treated in each school. The reported treatment coverage was consistently high between schools and across years and ranged from 69.7% to 91.3% and 86.2% to 96.5% in July 2010 and January 2012 SBD campaigns, respectively.

**Figure 2:**
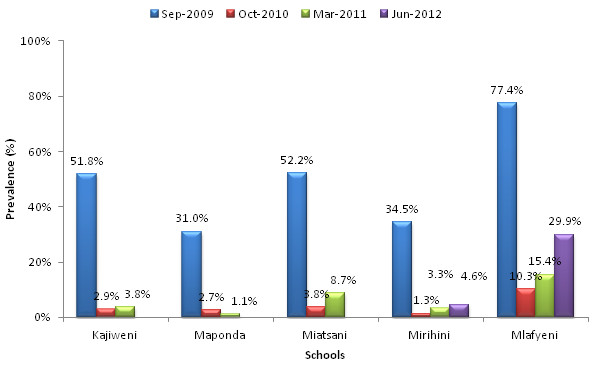
**Changes in prevalence of *S. haematobium *infection by school and year in Mwaluphamba Location, Mutuga District, Kwale County.** Overall, 505 (49.4%) of the 1022 children examined at baseline were found to have *S. haematobium* infection with Mlafyeni school having the highest rates (77.4%). The treatment significantly reduced the prevalence of *S. haematobium* infection in all schools. However, rebound in prevalence of *S. haematobium* infection was observed in most schools.

Figure 2 shows changes in prevalence of *S. haematobium* infection in the five schools during the study period. Significant differences in baseline prevalences of infection were seen among the five schools with Mlafyeni having the highest rates (77.4%). Except for one school (Maponda), varying levels of rebound in *S. haematobium* infection prevalences were seen after treatment. Table 3 provides a summary of the overall prevalence and intensities of urogenital schistosomiasis infection in the 5 study schools at the different survey time points. Application of the Chi-squared test for linear trend to test for linear association in the *S. haematobium* prevalences over the study period revealed a downward trend (P < 0.0001). Similarly, application of simple regression analysis showed a significant downward trend in the geometric mean egg count for the urogenital schistosomiasis infection across the years (P < 0.0001). The rebound observed in June 2012 follow-up survey did not significantly impact on this trend. Of 505 children with *S. haematobium* infection at baseline, 132 (26.1%) were also co-infected with STH infections mainly hookworms.

**Table 3:** ***Schistosoma haematobium *** **infection among children at baseline and follow-up surveys in Mwaluphamba Location, Mutuga District, Kwale County**

***S. haematobium *** **infection**	**Sep**	**Oct**	**Mar**	**Jun**	**P-value**
	**2009 (Baseline)**	**2010**	**2011**	**2012**	
No. positive	505 (49.4%)	45 (4.2%)	57 (5.9%)	72 (17.7%)	<0.0001
**Intensity of *S. haematobium *** **infection**					
Light infection, n (%)	279 (55.2%)	34 (75.6%)	40 (70.2%)	50 (69.4%)	0.081
Heavy infection, n (%)	226 (44.8%)	11 (24.4%)	17 (29.8%)	22 (30.6%)	
AM egg count (95% CI)	161.6(137.4-185.8)	94.9(29.9-159.9)	79.9 (39.5-120.3)	167.8 (22.6-313.0)	N/A
GM egg count (95% CI)	37.7 (31.8-44.5)	12.1(6.4-22.7)	17.6 (10.7-29.1)	16.8 (10.0-28.0)	<0.0001
**Co-infection of *S. haematobium *** **with other helminths**					
Any STH	132 (26.1%)	14 (31.1%)	16 (28.1%)	7 (9.7%)	
Hookworm	114 (22.6)	14 (31.1%)	16 (28.1%)	7 (9.7%)	
*A. lumbricoides*	12 (2.4%)	0 (0.0%)	0 (0.0%)	1 (1.4%)	
*T. trichiura*	10 (1.9%)	0 (0.0%)	1 (1.8%)	1 (1.4%)	

AM: arithmetic mean; GM: geometric mean; CI: confidence interval.

Light infection: 1–49 eggs/ml; Heavy infection: ≥ 50 eggs/ml.

AM and GM were calculated for infected individuals only.

**Figure 3:**
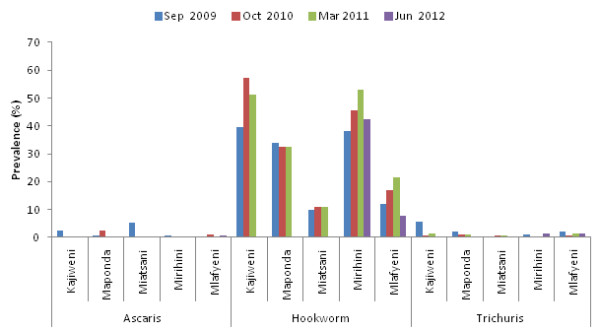
**Prevalence of STH infections in study schools at baseline and follow-up surveys in Mwaluphamba Location, Mutuga District, Kwale County.** The major STH infection in the area was hookworm but the once per year school-based deworming did not have a significant effect on the infection.

Figure 3 and Table 4 summarize the prevalences and intensities of STH infections in the 5 schools, respectively during the study period. Hookworm infection was found to be the most common STH infection in all the schools. All the *A. lumbricoides* and *T. trichiura* infections detected were light intensity according to WHO criteria whereas 98% of hookworm infections were also light intensity at baseline. There were no significant differences between females and males in prevalence of STH infections. The baseline prevalence of hookworm infection was 26.2% (range 9.7% to 39.6%) with two schools, namely Miatsani and Mlafyeni situated in the drier area in the North of the study location having lower prevalences (< 15%) compared to the other three schools towards the South (>30%). The school based treatment with albendazole and praziquantel, however, did not change both the overall prevalence and intensity of hookworm infection.

**Table 4:** Geometric and arithmetic means of STH infections at baseline and follow-up surveys in Mwaluphamba Location, Mutuga District, Kwale County

	**Sep 2009**	**Oct 2010**	**Mar 2011**	**Jun 2012**	**P-value**
**Geometric mean epg**^**a **^**(95% CI)**					
A. lumbricoides*	18.3 (14-23.9)	18.1 (9.2-35.7)	-	N/A	ND
Hookworm	98.8 (84.5-115.5)	80.8 (69.8-93.6)	102.8 (88.7-119.2)	121.9 (92.2-161.1)	0.6171
*T. trichiura*	27.4 (20.4-36.6)	25.1 (9.8-64)	31.8 (15-67.7)	28.8 (10.3-81)	0.3315
**Arithmetric mean epg**^**a **^**(95% CI)**					
*A. lumbricoides*	21.5 (14.9-28)	24 (2.7-45.3)	-	N/A	ND
Hookworm	269.5 (180.7-358.3)	221 (176.9-265)	277.2 (219.2-335.2)	291.2 (204.7-377.6)	0.5564
*T. trichiura*	33.3 (24.1-42.4)	31.2 (4.1-58.3)	49.5 (-3.7-102.7)	42 (4-80)	0.3437

^**a**^For infected individuals only.

CI: confidence interval.

*There was only one case of *Ascaris lumbricoides* in 2012*.* ND: Not Done, because of few data points.

## Discussion

The current study shows that both urogenital schistosomiasis and soil-transmitted helminths are significant public health problems in the rural areas of Kwale County, coastal Kenya. Among the soil-transmitted helminths, hookworm infection was the biggest problem. Generally, co-administration of praziquantel and albendazole helped treat urogenital schistosomiasis even though varying levels of rebounds in prevalences of *S. haematobium* infection were observed in most schools after each round of SBD. Mlafyeni school had relatively high baseline prevalence of *S. haematobium* infection (> 77%) at the baseline survey and a relatively big increase in schistosome infection prevalence (from 15.4% to 29.9%) was observed during the June 2012 follow-up survey six months after administration of the second round of SBD. However, the proportions of heavy intensity schistosome infections for the follow-up surveys before and after the second SBD in this school were not statistically different (34.8% in March 2011 Vs. 33.3% in June 2012). The reason for the large rebound in prevalence of schistosome infection in Mlafyeni is not clear especially since the first round of SBD resulted in quite impressive decline in both prevalence and intensity of schistosomiasis in all schools. Nonetheless, the slight increases seen in most schools during the follow-up surveys suggest that once per year co-administration of praziquantel may not be adequate for the complete control of urogenital schistosomiasis in the area particularly for schools with relatively higher baseline prevalence rates. Small-scale heterogeneity of parasitic infections has important implications for control and it has been suggested that more sensitive diagnostic tools or rigorous sampling approaches are needed to select endemicity settings with high fidelity [10]. Mlafyeni school may be considered to be a local hotspot contributing to high transmission of urogenital schistosomiasis in the area. Schools with such high prevalences should be identified and considered for increased frequency of treatment under the national control programmes. The WHO guidelines for preventive chemotherapy in human helminthiasis categorize areas with schistosome infection prevalences of ≥ 50% among school-age children as high-risk communities [11]. In such areas, the guidelines recommend once per year treatment of all school-age children including adult populations that may be considered to be at risk, such as persons engaged in occupations that expose them to contact with schistosome infested waters. Where the entire population is considered to be at risk, a community-wide treatment campaign should be considered.

Matuga District, as well as other districts in Kwale County, had previously received several rounds of community-wide distribution of albendazole plus DEC through the National Programme for Elimination of Lymphatic Filariasis (NPELF) before implementation of the current study, albeit not in consecutive years. The last MDA against LF was conducted in December 2008 roughly ten months before the baseline survey in September 2009. The administration of albendazole in MDAs for LF may partly explain why the STH infections in the area were light intensity. Nonetheless, hookworm was identified as a significant STH infection in children in the study area and co-administration of praziquantel and albendazole had little effect on this infection even though reported treatment coverage was consistently high (>86%). With the overall baseline prevalence for any STH infection among school-age children being between ≥20% and <50% in the area, the WHO guidelines categorizes the area as low-risk for STH and recommends treatment of all school-age children once each year [11]. The guidelines also do recommend inclusion of adult populations considered to be at risk in the treatment. We have previously shown that adults may be an important reservoir of helminthic infections in this area particularly hookworm and urogenital schistosomiasis [8]. The apparent lack of decline in prevalence of hookworm infection in children after each treatment is therefore noteworthy. The area has very low sanitation as found through a demographic survey which revealed that only 21.6% of the households had latrines. Observations indicated that the majority of the community members defecate in the bushes and other areas around the homes. A cross-sectional survey by a different team in a neighbouring district also in Kwale County found hookworm infection to be the major STH in school-age children [12]. The current study, therefore, underscores our earlier call for design of other treatment strategies and complementary interventions for effective control of hookworm infection in rural areas of Kwale County where the adult population may be an important reservoir of the infection and thus should be considered for treatment [8].

Challenges in acquiring funding to support NTD programmes may obviously jeopardize integration efforts. For example, the LF programme had planned to conduct MDA in October 2010 but that did not happen until May 2011. Unfortunately, the treatment coverage among residents of Mwaluphamba Location for the MDA against LF conducted in May 2011 was very low (46.6%), which was mainly attributed to inadequate community mobilization. A carefully coordinated or integrated NTD programme for the control and elimination of LF, STH and urogenital schistosomiasis in Kwale and other areas of coastal Kenya is recommended. Further, implementation research is required to determine optimal delivery strategies able to reach all sections of the endemic population so as to attain recommended coverage rates.

The study had several limitations. Firstly, it was conducted in 5 schools in one administrative location of Matuga District, so the findings may not be representative of the entire Kwale County. Also, only 2 schools were included in the endline follow-up survey thus not allowing measurements to be conducted in all the study schools. The second round of praziquantel/albendazole school-based treatment was not given exactly after one year since the first treatment which makes it difficult to interpret the results. The delay was caused by delayed mass drug treatment against LF by the national LF programme. This, however, may reflect the real challenges and ensuing logistical complexities that may arise for integrated NTD programmes if one intervention is delayed. A previous report has indeed underscored technical and managerial challenges faced when integrating control for different NTDs and the need for their appreciation at the outset, so as to be able to manage expectations of stakeholders [13].

There was a slightly lower prevalence of hookworm infection at baseline compared to the post-treatment surveys, but we believe this is an artifact probably due to lower technical competency of the laboratory team at baseline. The technical competency of the laboratory technicians seems to have improved during subsequent surveys. This observation, however, underscores the importance of training technical survey teams for accuracy of data during NTD surveys. Most countries in the sub-Saharan African region have developed NTD master plans and are currently preparing to implement control programmes. The first of such programmes will be preventive chemotherapy NTD programmes including SBD. We propose that thorough practical training sessions be given to district level laboratory technicians supporting such programmes in efforts to improve data quality. The laboratory tests currently employed for epidemiological surveys of STH and urogenital schistosomiasis under NTD control programmes are not part of routine laboratory diagnostic assays in most health facilities. This means that most technicians do not have the required experience in Kato-Katz and urine filtration techniques and hence the need for practical training during the implementation of such programmes.

Further studies are required to determine optimal treatment frequency in areas such as Matuga District and the studies could also include evaluating the role of complementing preventive chemotherapy with health education and promotion particularly focused to construction of latrines and their use for disposal of human excreta so as to decontaminate the environment, thereby reducing community transmission rates.

## Conclusions

The results of the current study suggest once per year treatment may not be adequate preventive chemotherapy strategy against hookworm and *S. haematobium* infections in areas where transmission is high and sanitation coverage is low, such as in rural areas of Kwale County. Our intervention failed to reduce the prevalence of hookworm and rebounds in rates of urogenital schistosomiasis were observed. Thus there is an urgent need to consider alternative treatment regimens so as to effectively control the infection in the area. Since these infections were previously shown to be high in adults in this area [8], it is critical to consider expanding preventive chemotherapy to include all community members in the ongoing intervention programmes. For sustainable NTD control in this area, it is important to also find ways of engaging the communities in construction of household latrines as well as hygiene behavioral change so as to improve community level sanitation.

## Competing interests

The authors declare that they have no competing interests.

## Authors’ contributions

SMN, CNW and MJB participated in the conception and design of the study. FMM supervised specimen collection and laboratory examinations. KKN provided clinical oversight during the study. SMN drafted the manuscript whereas CNW, MJB and MTM revised it critically for intellectual content. All authors read and approved the final manuscript. SMN is the guarantor of the paper.
